# Perceived Stress, Risk Factors, and Learning Challenges among Nursing Students

**DOI:** 10.1192/j.eurpsy.2026.12105

**Published:** 2026-07-31

**Authors:** A. Aloui, A. Chouchane, N. Ganoun, I. Kacem, M. Bouhoula, M. Makhloufi, H. Kalboussi, O. El Maalel, S. Chatti

**Affiliations:** Department of Occupational Medicine, Farhat Hached University Hospital, Sousse, Tunisia

## Abstract

**Introduction:**

The demanding academic environment is a potential source of stress for nursing students, and the inability to cope effectively often exacerbates this burden.

**Objectives:**

To assess the level of perceived stress among nursing students, identify its risk factors and manifestations, and evaluate its impact on their learning.

**Methods:**

A descriptive cross-sectional study was conducted among students at a private nursing institute in Sousse, using a questionnaire administered between February and March 2023. Perceived stress was measured using the Perceived Stress Scale (PSS).

**Results:**

The study population was predominantly female (75.7%) with a sex ratio of 0.32. The mean age was 21.5 years, and second-year students represented 66.7% of the sample. The average perceived stress score was 27.21 ± 5.71 (range: 18–35). Nearly two-thirds of participants (60.7%) experienced persistent stress (score > 26). The main stressors related to theoretical learning were examinations (36.7%) and the need to adapt to new academic demands and heavy workloads (36.3%). Regarding clinical learning, the most frequently reported stressors were fear of failure (65.7%) and deteriorating clinical training conditions (52%). Somatic manifestations included sleep disturbances (72.3%) and headaches (72%). Psychological manifestations included decreased motivation (72%) and mood instability (65%). Stress negatively impacted learning, as 88% of students reported increased delays and absenteeism from classes and internships, while 66% indicated difficulty in understanding courses, case studies, and clinical activities when stressed. Suggested coping strategies included systematic screening (66.7%) and stress management training (64%).

**Conclusions:**

The moderate stress levels reported by nursing students highlight the need for structured stress management programs and appropriate support systems. As future healthcare providers, early interventions during academic training and clinical practice are essential to reduce stress and strengthen coping abilities.

**Disclosure of Interest:**

None Declared

